# Lactose Malabsorption and Lactose Intolerance in Children with Inflammatory Bowel Diseases

**DOI:** 10.1155/2019/2507242

**Published:** 2019-12-01

**Authors:** Martyna Jasielska, Urszula Grzybowska-Chlebowczyk

**Affiliations:** Department of Pediatrics, Medical University of Silesia, Katowice, Poland

## Abstract

**Background:**

Insufficient vitamin D and calcium intake associated with the restricted intake of milk and dairy products can lead to poor health outcomes like malnutrition and abnormal bone mineralization. The aim of the study was to estimate the prevalence of primary and secondary lactose intolerance in children with IBD.

**Methods:**

The study included 107 patients (mean age 14.07 ± 3.58 years; 46.7% boys) which includes 43 patients with Crohn's disease (CD), 31 with ulcerative colitis (UC), and 33 children with functional abdominal pain (AP-FGID). We analysed the result of the hydrogen breath test with lactose loading, two single nucleotide polymorphisms of the *LCT* gene (LCT-13910CC and LCT-22018GG). The results were analysed with MedCalc Statistical Software.

**Results:**

Adult-type hypolactasia (ATH) was found in 31% of patients with IBD and 42.4% of AP-FGID (*p* = 0.2). Lactose malabsorption (LM) was found in 27.9% of patients with CD, in 22.6% with UC, and in 24.2% with AP-FGID (*p* = 0.8). Lactose intolerance (LI) was diagnosed in a similar percentage of patients in each group (*p* = 0.9). Secondary LI in IBD patients does not depend on the location, duration, and activity of the disease and the number of relapses (*p* > 0.05). The median time of lactose-free diet in CD was 10 months and in CU 24 months.

**Conclusions:**

The incidence of LI, LM, and ATH does not differ among children with IBD from the population.

## 1. Introduction

Adult-type hypolactasia (ATH) is the most common cause of maldigestion and malabsorption of lactose [1, 2]. It is caused by genetically determined variability of enzymatic activity of lactase during human life. Lactase activity is highest at birth and declines after weaning, so in adolescents and adults with ATH, lactase activity reaches 5-10% of that occurring in infancy [1, 3]. The age at which this decline starts depends on ethnic and geographical origin, as well as the usual intake of dairy products in the diet in a population. Among Europeans, two single nucleotide polymorphisms (SNP) were described upstream of the *LCT* gene; LCT-13910CC and LCT-22018GG have been associated with ATH [4]. Genotypes LCT-13910CT, LCT-13910TT, LCT-22018GA, and LCT-22018AA determine sufficiently high lactase activity over entire life. In addition, it was proven that the genotypes of both variants exhibit almost complete compliance; patients with the LCT-13910CC genotype also had the LCT-22018GG genotype, and patients with the LCT-13910TT genotype has the LCT-22018AA genotype [3]. In Poland, the incidence of lactose malabsorption (LM), assessed by the hydrogen breath test, varies from 17.4 to 37.5%, while the incidence of the LCT-13910CC genotype responsible for ATH was estimated at 30-31.5% [5–7]. Typical symptoms of lactose intolerance (LI) are diarrhoea, bloating, abdominal pain, nausea, and vomiting. Their occurrence results from bacterial fermentation of undigested lactose by intestinal bacteria *Bifidobacterium*, *Lactobacillus*, and *Streptococcus*, having the enzyme beta-galactosidase.

Secondary lactose malabsorption and intolerance are caused by damage of the small intestinal mucosa. One of the reasons of such damage is Crohn's disease (CD). Secondary LM is usually transient and lasts a few weeks [2].

The influence of dairy products on pathogenesis and the course of inflammatory bowel diseases (IBD) are unclear. A prospective cohort study on the European population showed that consuming dairy products reduces the risk of Crohn's disease (CD) [8]. There are also reports that consumption of 1.25 litres of milk per week reduces the symptoms of ulcerative colitis (UC) [9]. The incidence of lactose intolerance in patients with inflammatory bowel diseases has already been the subject of investigation, but the obtained results are not conclusive. In a meta-analysis conducted by Szilagyi et al., LM in adult patients with Crohn's disease was more frequent than in the control groups, while in the patients with ulcerative colitis, this incidence does not differ from the control [10]. However, the current research concerns mainly adults, and there is little data on the incidence of lactose malabsorption and lactose intolerance among children with IBD.

## 2. Aims

The aim of the study was to estimate the incidence of adult-type hypolactasia, lactose malabsorption, and lactose intolerance in children with inflammatory bowel diseases.

## 3. Materials and Methods

The analysis included a total of 107 patients aged from 5 to 18 years (mean 14.07 ± 3.58, median 15.6 years); 46.7% were boys. The examined children were patients at the Department of Gastrology, Upper Silesian Children's Health Centre, Katowice, in the years 2016-2017. Subjects were divided according to their diagnosis: Crohn's disease (CD, *n* = 43), ulcerative colitis (UC, *n* = 31), and functional abdominal pain (AP-FGID, *n* = 33) (Table 1).

The diagnosis of IBD was based on the modified Porto criteria [11]. 15 patients (20.2%) were newly diagnosed with IBD. The AP-FGID was diagnosed on the basis of 4th Rome criteria [12]. The examined groups showed no statistically significant differences in terms of age and sex (*p* > 0.05).

All patients were subjected to the hydrogen breath test (HBT). The test was conducted in the morning, after an 8 to 12-hour fasting. A Gastrolyzer Gastro+ (SynecPOL, Poland) was used to measure hydrogen concentration in exhaled air. Measurements were carried out every 30 minutes for 3 hours after oral ingestion of lactose (1 g lactose/kg body weight, maximum 25 g). Lactose malabsorption was diagnosed, if in any of the measurements the hydrogen concentration in the exhaled air increased ≥20 ppm over the baseline value. The occurrence of clinical symptoms such as diarrhoea, bloating, abdominal pain, nausea, and vomiting within 12 hours of the start of the test was diagnosed as lactose intolerance. The test was usually performed after resolution of acute symptoms, at an interval of at least 4 weeks from antibiotic therapy.

In all subjects, peripheral blood was drawn. Genomic DNA was isolated from the blood in the Spin Protocol (QIAGEN, Germany). The GenoType LCT test (Hain Lifescience GmbH, Germany) was used for detection of the *LCT* gene polymorphisms, which is a strip test.

The primary lactose intolerance was diagnosed in patients with two single nucleotide polymorphisms of the *LCT* gene connected to ATH (genotypes LCT-13910CC and LCT-22018GG) and who had clinical symptoms of lactose intolerance after oral ingestion of lactose in HBT. Secondary lactose intolerance was diagnosed in children with negative genetic predisposition to ATH (genotypes LCT-13910CT, LCT-13910TT, LCT-22018GA, and LCT-22018AA) and with positive HBT after oral ingestion of lactose (only when clinical symptoms of lactose intolerance occurred) [1].

Also, the medical history and data from medical records of all the patients were taken. IBD patients were also evaluated for the clinical expression of the disease: the location of the disease (the Paris classification) and the disease activity (PCDAI/PUCAI) [13–15].

Statistical analysis was conducted using the procedures available in licensed MedCalc Statistical Software version 17.7 (MedCalc Software bvba, Ostend, Belgium; 2017). The character of the distribution of quantitative variables was verified by the Shapiro-Wilk test. The evaluation of intergroup differences for quantitative variables was based on Student's *t*-test (or ANOVA), in the case of variables with normal distribution, or Mann-Whitney *U* test (or Kruskall-Walis), in the case of variables with a nonnormal distribution. Intergroup differences for categorical variables were evaluated by a chi-square test or Fisher's exact test. *p* < 0.05 was considered statistically significant.

The protocol of the research was approved by the Bioethical Committee of the Silesian Medical University in Katowice, Poland. Research was carried out with funds from the Medical University of Silesia in Katowice: KNW-2-026/D/5/N and KNW-2-K36/D/6/N.

## 4. Results

### 4.1. Location and Activity of IBD

The mean duration of CD and UC was similar and was, respectively, 21.27 months (median 13 months, range 1-108 months) and 21.72 months (median 12 months, range 0.5-96 months). The age of diagnosis, the location of the disease, and the behaviour of inflammatory bowel diseases are classified on the basis of Paris classification [13] (Table 2).

A moderate disease was most commonly diagnosed in 20 children with CD (46.5%). A mild disease was diagnosed in 18 children (41.9%) and severe disease in 5 children (11.6%). In ulcerative colitis, mild disease was diagnosed in 14 children (45.2%), similar to the moderate disease in 14 children (45.2%). A severe disease was diagnosed only in 2 children (6.4%). One child was diagnosed as inactive (3.2%). The distribution of disease activity did not differ in the IBD groups (*p* > 0.05).

### 4.2. Lactose Intolerance

LI was diagnosed in a similar percentage of patients in each group: CD (23.2%), UC (22.6%), and AP-FGID (21.2%) (*p* = 0.9). Secondary lactose intolerance was most often diagnosed in the group of children with CD (9.3%) (vs. UC (3.2%) and AP-FGID (6%)); however, this difference was also not statistically significant (*p* = 0.8). LM was found in 19 patients with IBD (25.6%), including 12 patients with CD (27.9%) and 7 patients with UC (22.6%). In the AP-FGID group, lactose malabsorption concerned 24.2% of subjects; these differences were not statistically significant (*p* = 0.8) (Table 3).

SNP of the promoter region of the *LCT* gene associated with ATH were found in 31% patients from the IBD group and 42.4% with AP-FGID; this difference was not statistically significant (*p* = 0.25). In the IBD group, ATH was more frequent in patients with UC (38.7%) than in patients with CD (25.6%); however, this difference was also not statistically significant (*p* = 0.2). In all groups, a full agreement was found between the genotypes in the loci LCT-22018 and LCT-13910 (Table 4).

Four patients with CD and one with UC were diagnosed with secondary LI. The average duration of secondary LI following a low-lactose or lactose-free diet in these patients was 3.5 months (range 2-6 months). The occurrence of secondary LI among children with IBD did not depend on the location, duration, and activity of the disease (measured in the PCDAI and PUCAI) and the number of relapses (*p* > 0.05). The results of the histological examination of duodenal biopsies in children with secondary lactose intolerance were normal; no inflammation or villus atrophy was found.

### 4.3. Elimination Diet

A lactose-free or low-lactose diet was followed by all children diagnosed with LI (17 children with IBD and 8 from the control group). The median time of adhering to this diet by patients with CD is 10 months (range 1-108 months) and with UC, 24 months (range 2-96 months). Four patients with primary lactose intolerance (2 with CD and 2 with UC) followed a low-lactose diet for a longer period (70-108 months).

## 5. Discussion

In this research in the group with Crohn's disease, and in the group with ulcerative colitis, the incidence of LI was similar to that in the AP-FGID and was 23.2%, 22.6%, and 24.2%, respectively. Secondary lactose intolerance in IBD was diagnosed in 6.7% of patients, and its occurrence was not dependent on such factors as location, activity, and duration of the disease or the number of relapses in the past. However, the observed group of children was too small to make any conclusions. Patients with IBD are a group at a high risk of malnutrition, deficiency of macro- and micronutrients and vitamins, and disorders of bone mineralization. Unnecessary elimination of dairy products from the diet may aggravate these deficiencies. In order to meet the above problems, the aim of the study was to assess the prevalence of LI in patients with IBD. In the available Polish and worldwide literature, the number of studies assessing the incidence of LI in this group of patients is limited. The currently available researches more often assess the incidence of lactose malabsorption than lactose intolerance and more commonly concern the adult population. Compared to our study, there was a slightly higher incidence of lactose intolerance (about 40%) among patients with IBD assessed by Pawłowska et al. among children with various diseases of the gastrointestinal tract; however, the group of children with IBD included only 25 patients [16].

In several Polish studies, the incidence of the LCT-13910CC genotype associated with ATH in healthy people was estimated at 30-31.5% [7, 17], which was a comparable incidence to that obtained in the whole group of patients with IBD (31%), while slightly less than that in the group of children with AP-FGID (42.4%); the difference was not significant. In all patients, the presence of LCT-13910CC and LCT-22018GG genotypes was consistent. Only a few studies assessing the prevalence of the LCT-22018GG genotype in the Polish population are available. In Adler et al.'s study, the incidence of the LCT-22018GG genotype among healthy adults was 30% and among CD patients was 34% and was also consistent with that of the LCT-13910CC genotype [17]. Similarly, Büning et al. in Germany did not find differences in the incidence of LCT-22018GG and LCT-13910CC genotypes between patients with IBD, their first-degree relatives, and healthy volunteers [18].

The unresolved issue is the effect of different SNP of the promoter region of the encoding gene of the lactase on the incidence of IBD. In New Zealand, Nolan et al. demonstrated with a large group of patients that T-allele in locus LCT-13910, responsible for lactase persistence, occurs more often in patients with Crohn's disease [19]. These researchers tried to explain the increased risk of developing CD, in those consuming dairy products, the fact that *Mycobacterium avium* subsp. *paratuberculosis* (MAP) may be present in dairy products. MAP causes ruminant intestinal disease, called John's disease, where the symptoms are similar to those occurring in Crohn's disease, so this bacterium was considered one of the environmental factors involved in the development of the CD [19]. However, this theory has not been confirmed so far [20]. Also, Elguezabal et al. also did not prove any connection between the genotype LCT-13910CC and IBD. However, it has been noticed that T allele is less frequent in patients with UC [21]. Similarly, in the examined group, in patients with UC, LCT-13910TT genotype occurred more often than in other groups; however, this difference was not statistically significant.

LM in the Polish population concerns 17.4-37.5% of people [5, 6, 22]. The expression of the lactase in the intestine in people with ATH decreases with age. LM concerns 11-17.4% of children under 15 years of age and 22% of adolescents aged 15-19 [5, 22, 23]. Among the studied patients, LM occurred slightly more often than in the mentioned studies. LM was similarly frequent in each of the studied groups, with slightly more frequent cases in patients with CD (27.9%). In a meta-analysis conducted by Szilagyi et al., LM in patients with Crohn's disease was more frequent than that in the control groups [10]. Also in the study by Wiecek et al. assessing the activity of disaccharidases in the small intestine biopsy in children with IBD, decreased lactase activity was found in 33% of patients with CD and 20% with UC [24]. On the other hand, the study by Pfefferkorn et al. did not show an increased incidence of decreased lactase activity in the intestine of patients with IBD in relation to the control but found that 67% of patients with lactose malabsorption had normal small intestine biopsies (without villous atrophy and inflammatory infiltrates in the mucous membrane). In the same study, 18% of patients with normal lactase activity have mild to severe inflammation and villous atrophy [25]. All patients with IBD and LM or LI (also secondary) in the conducted study had a normal duodenal biopsy.

In our study, lactose intolerance was diagnosed in a percentage (CD—23.2%, UC—22.6%, and AP-FGID—24.2%) very similar to the percentage of all positive breath tests (CD—27.9%, UC—22.6%, and AP-FGID—21.2%). In the current studies, most patients with ATH have positive hydrogen breath test, but without symptoms of lactose intolerance [1, 2, 22]. In a research on the Polish healthy population carried out by Marasz, 48% of patients had LM, and 15.5% had LI [22]. On the other hand, very similar results to ours were found in the study by Pawłowska et al. among children with IBD and FGID [16]. It requires further research whether patients with gastrointestinal tract diseases with ATH more often develop symptoms of lactose intolerance than healthy ones.

In current research studies, there is an increased risk of LM and LI associated with the location of inflammatory changes in the small intestine and the terminal ileum with colon involvement, while the colonic location alone was irrelevant; however, this study concerned mainly adults [10]. In the assessed group of patients with CD, the location of the disease had no effect on the incidence of both LM and LI. The disease activity in the study group also had no effect on LM [10]. In contrast, von Tirpitz et al. in adult patients with CD found out that higher disease activity measured on the CDAI scale (Crohn's Disease Activity Index) was associated with a higher prevalence of lactose digestive disorders [26].

All patients with LI followed a low-lactose or lactose-free diet. Even patients who had followed the elimination diet for a long time, after an increased load of lactose, experienced symptoms of intolerance. According to the current studies, the majority of patients with lactose intolerance may consume up to 12.5 g of lactose (equivalent to 245 ml of milk) per day without any symptoms of intolerance [27]. The ingestion of a gradually increasing amount of lactose into the diet results in a reduction of clinical symptoms of intolerance, alteration of the intestinal microflora, increased bacterial beta-galactosidase activity in the large intestine, and a decrease in exhaled hydrogen concentration in the HBT after oral ingestion lactose [4, 28, 29]. In the studied group, patients did not repeat the HBT; however, the occurrence of clinical symptoms after consumption of products containing lactose indicates a lack of adaptation. The research protocol did not include diet control (e.g., in the form of a diary); information about the diet was collected during the interview with the patient and his/her parents, which means that they may not be fully reliable and which is a weakness of this study. However, it should be noted that in a study from Israel, it was proven that only 1/3 of patients with lactose intolerance developed adaptation and returned to a normal diet in the 3 years of observation [30].

## 6. Conclusions

To our knowledge, this is the first study which simultaneously estimates the incidence of both genotypes associated with ATH, LM, and LI in paediatric patients with IBD. In our study, the incidence of SNP of a promoter region of *LCT* gene, lactose malabsorption, and intolerance does not differ among children with inflammatory bowel diseases from the population. The development of knowledge about the prevalence of lactose malabsorption and LI may result in important implications in the diagnostic and healing process of IBD patients, preventing unnecessary implementation of elimination diets and thus contributing to the prevention of future complications of the disease, including malnutrition and the occurrence of calcium phosphate metabolism disorders.

## Figures and Tables

**Table 1:** Demographic characteristics of the studied groups.

Group	*N*	Age (years)			Boys *N* (%)
		Average	SD	Range	
CD	43	15.14	2.36	7-17.9	20 (46.5%)
UC	31	13.84	4.22	5-18	19 (61.2%)
AP-FGID	33	12.9	3.92	5.5-18	11 (33.3%)

**Table 2:** Paris classification.

	Symbol	Description	*N* (%)
Crohn's disease			

Age of diagnosis	A1a	0–<10 years	6 (14%)
	A1b	10–17 years	35 (81.4%)
	A2	17-40 years	2 (4.6%)
	A3	>40 years	—

Location	L1	Distal 1/3 ileum (±limited cecal disease)	20 (46.5%)
	L2	Colonic	6 (13.9%)
	L3	Ileocolonic	13 (30.3%)
	L4a	Upper disease proximal to ligament of Treitz	12 (27.9%)
	L4b	Upper disease distal to ligament of Treitz and proximal to distal 1/3 ileum	6 (13.9%)

Behaviour	B1	Nonstricturing Nonpenetrating	31 (72.1%)
	B2	Stricturing	9 (20.9%)
	B3	Penetrating	3 (7%)
	p	Perianal disease modifier	7 (16.3%)

Growth	G0	No evidence of growth delay	31 (72.1%)
	G1	Growth delay	12 (27.9%)

Ulcerative colitis			

Extent	E1	Ulcerative proctitis	6 (19.4%)
	E2	Left-sided UC (distal to splenic flexure)	10 (32.2%)
	E3	Extensive (hepatic flexure distally)	6 (19.4%)
	E4	Pancolitis (proximal to hepatic flexure)	9 (29%)

Severity	S0	Never severe	24 (77.4%)
	S1	Ever severe	7 (22.5%)

**Table 3:** Distribution of the incidence of ATH, lactose malabsorption, and lactose intolerance in the studied groups.

		CD [43] *N* (%)	UC [31] *N* (%)	AP-FGID [33] *N* (%)	*p*
Adult-type hypolactasia		11 (25.6%)	12 (38.7%)	14 (42.4%)	0.2

Lactose malabsorption		12 (27.9%)	7 (22.6%)	8 (24.2%)	0.8

Lactose intolerance	All	10 (23.2%)	7 (22.6%)	7 (21.2%)	0.9
	Primary	6 (13.9%)	6 (19.4%)	5 (15.2%)	0.8
	Secondary	4 (9.3%)	1 (3.2%)	2 (6%)	0.8

**Table 4:** Results of single nucleotide polymorphisms of the LCT gene in loci LCT-13910 and LCT-22018.

Group [*N*]	LCT-13910			LCT-22018		
	C/C^∗^ *N* (%)	C/T *N* (%)	T/T *N* (%)	G/G^∗^ *N* (%)	G/A *N* (%)	A/A *N* (%)
CD [43]	11 (25.6%)	18 (41.9%)	14 (32.5%)	11 (25.6%)	18 (41.9%)	14 (32.5%)
UC [31]	12 (38.7%)	14 (45.2%)	5 (16.1%)	12 (38.7%)	14 (45.2%)	5 (16.1%)
AP-FGID [33]	14 (42.4%)	12 (36.4%)	7 (21.2%)	14 (42.4%)	12 (36.4%)	7 (21.2%)

^∗^Genotypes responsible for ATH.

## Data Availability

The statistical analysis and database used to support the findings of this study may be released upon application to the Department of Pediatrics, Medical University of Silesia, which can be requested thru the corresponding author.
